# Enhanced hydrogen uptake of dihydrogen complex *via* porous materials support†

**DOI:** 10.1039/d4ra01182a

**Published:** 2024-04-09

**Authors:** Kaiji Uchida, Shunya Tanaka, Shuta Adachi, Hiroaki Iguchi, Ryota Sakamoto, Shinya Takaishi

**Affiliations:** a Department of Chemistry, Graduate School of Science, Tohoku University Sendai Miyagi 980-8578 Japan shinya.takaishi.d8@tohoku.ac.jp; b Tokyo Metropolitan Industrial Technology Research Institute 2-4-10 Aomi Koto Tokyo 135-0064 Japan; c Department of Chemistry, Faculty of Science, Tohoku University 6-3 Aza-Aoba, Aramaki Sendai 980-8578 Japan; d Physical and Chemical Research Infrastructure Group, RIKEN SPring-8 Center, RIKEN Sayo Hyogo 679–5198 Japan

## Abstract

This research focuses on enhancing H_2_ adsorption by using the [Mo(PCy_3_)_2_(CO)_3_] complex supported on porous materials such as silica gel and mesoporous carbon. The study reports a significant increase in hydrogen adsorption capacity, reaching up to 9.3 times that of the bulk complex. This improvement suggests that using mesoporous materials as supports for the [Mo(PCy_3_)_2_(CO)_3_] complex enhances the accessibility of H_2_ gas to its open-metal sites.

## Abstract 2

This study shows using [Mo(PCy_3_)_2_(CO)_3_] complex supporting on the porous materials boosts H_2_ adsorption up to 9.3 times by enhancing gas access to metal sites.

## Graphical abstract

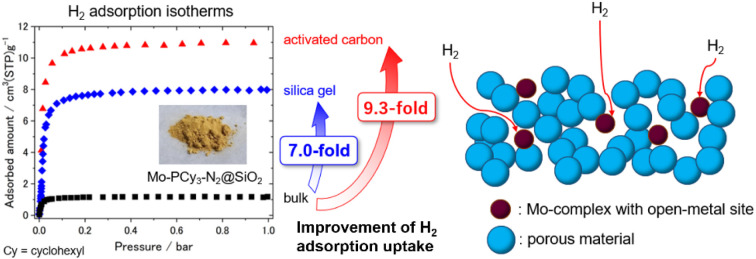

Dihydrogen (H_2_)-related technologies have been extensively studied due to their potential as an energy carrier, attributed to their high energy density (120 MJ kg^−1^*vs.* 44.5 MJ kg^−1^ for gasoline) and environmentally friendly CO_2_-free power production. However, H_2_ storage remains one of the main challenges in this field. Various physisorptive and chemisorptive materials, including metal–organic frameworks (MOFs),^1–7^ covalent organic frameworks (COFs),^8^ chemical hydrides,^9–11^ and hydrogen-absorbing alloys,^12^ have been intensively studied. These can be broadly categorized into physisorptive and chemisorptive mechanisms, each with its own advantages and disadvantages. Physisorptive materials exhibit a small adsorption enthalpy (|Δ*H*°| < 15 kJ mol^−1^), requiring low temperatures, such as those of liquid nitrogen (77 K), for effective H_2_ storage. Conversely, chemisorptive materials form metal hydrides accompanied by the cleavage of the H–H bond, resulting in a large adsorption enthalpy (typically, |Δ*H*°| > 70 kJ mol^−1^), which leads to irreversible adsorption and extremely slow kinetics at room temperature. To achieve reversible H_2_ adsorption/desorption under ambient conditions, an intermediate adsorption enthalpy (20 kJ mol^−1^ < |Δ*H*°| < 50 kJ mol^−1^) is necessary.

In recent years, Kubas interactions, which involve two types of orbital interactions: σ-donation and π-backdonation, have gained attention as a new class of adsorbents. The Kubas interaction, named after the researcher who first observed a side-on (η^2^) type M–H_2_ bond in dihydrogen complexes, is expected to result in larger adsorption enthalpies (|Δ*H*°|) compared to standard physisorption, owing to its unique orbital interactions. Simultaneously, while the H–H bonding weakens compared to free H_2_ molecules, it is still maintained, allowing Kubas interaction to afford an intermediate |Δ*H*°| between physisorption and chemisorption.

Over 100 types of dihydrogen complexes have been synthesized^13–18^ since the first complex, [Mo(PCy_3_)_2_(CO)_3_] (PCy_3_ = tricyclohexylphosphine), was reported by Kubas in 1984.^19^ However, research on hydrogen adsorption in solid-state has been quite limited. We have recently reported the reversible adsorption of H_2_ at ambient temperatures using the solid-state molybdenum complex, [Mo(PCy_3_)_2_(CO)_3_].^20^ The |Δ*H*°| for H_2_ adsorption was estimated to be approximately 50 kJ mol^−1^, positioning it between physi- and chemi-sorption. This characteristic makes it a viable candidate for hydrogen storage material, as it allows for reversible hydrogen adsorption and desorption at room temperature. However, this complex is a non-porous mononuclear complex, and its saturation adsorption capacity is quite low (*ca.* 1 cm^3^ (STP) g^−1^). Assuming that one molecule of [Mo(PCy_3_)_2_(CO)_3_] (molecular weight = 740.84) adsorbs one H_2_ molecule, the estimated saturated adsorption capacity is calculated to be 30.25 cm^3^ (STP) g^−1^. However, the actual measured saturated adsorption capacity is only 4% of this estimated value. This discrepancy is attributed to the fact that H_2_ adsorption occurs only on the surface of the particles. To utilize the open-metal sites for H_2_ adsorption, recent research has focused on facilitating Kubas interactions within MOFs using low-valent V^II^ or Cu^I^ open metal sites. However, among the vast library of MOFs, only three examples, namely Cu(i)-MFU-4l (27 kJ mol^−1^),^21^ V^II^-MOF (22 kJ mol^−1^),^4^ and NU-2100 (32 kJ mol^−1^)^22^ have been shown to exhibit |Δ*H*°| > 20 kJ mol^−1^. This is thought to be due to the extreme difficulty in designing MOFs with low-valent open metal sites that can effectively interact with hydrogen molecules.

In this study, we utilized mesoporous materials as supports for the metal complex [Mo(PCy_3_)_2_(CO)_3_] to improve the accessibility of H_2_ gas to its open-metal sites. We selected mesoporous materials: silica gel (wako-gel 60N) and mesoporous carbon (CNOVEL-MH). For comparison, microporous carbon (MSC-30) was also employed as a support material. The hydrogen adsorption uptake increased significantly when the complex was supported on these mesoporous materials, reaching up to 9.3 times that of the bulk sample. The thermodynamic parameters upon H_2_ adsorption were mostly identical to those of the unsupported complex, indicating that these parameters are intrinsic to the nature of the molecule and are not significantly affected by the molecular packing in the solid.

The mesoporous-material-supported [Mo(PCy_3_)_2_(CO)_3_] complexes were synthesized using a technique in which one component of the precursors (Mo(cht)(CO)_3_; cht = cycloheptatriene) was pre-adsorbed within a porous material, followed by the addition of the reactant (PCy_3_), as illustrated in Fig. 1(a). The detailed synthetic procedure is provided in the ESI.† Two types of support materials, silica gel and mesoporous carbon, were employed. Fig. 1(a) provides an example using silica gel. The successful synthesis of the complex in the presence of the support material was confirmed by infrared spectroscopy (IR) measurements, with results shown in Fig. 1(b). Immediately after synthesis, the nitrogen complex [Mo(PCy_3_)_2_(CO)_3_(N_2_)] (Mo-PCy_3_-N_2_) was supported. The stretching vibrations of C–H, N–N, and C–O were observed at the same positions as in the bulk complex, confirming that the complex was synthesized in the presence of the support material. The powder sample of the mesoporous materials-supported [Mo(PCy_3_)_2_(CO)_3_(N_2_)] was placed into an adsorption tube, and N_2_ was removed by dynamic vacuum for 12 hours at 100 °C prior to the measurement. The adsorption measurement results at 313 K are presented in Fig. 2. The graph clearly demonstrates that the hydrogen adsorption capacity of the supported materials significantly surpasses that of the bulk complex, with a 7.0-fold improvement for silica gel and a 9.3-fold improvement for activated carbon.

**Fig. 1:**
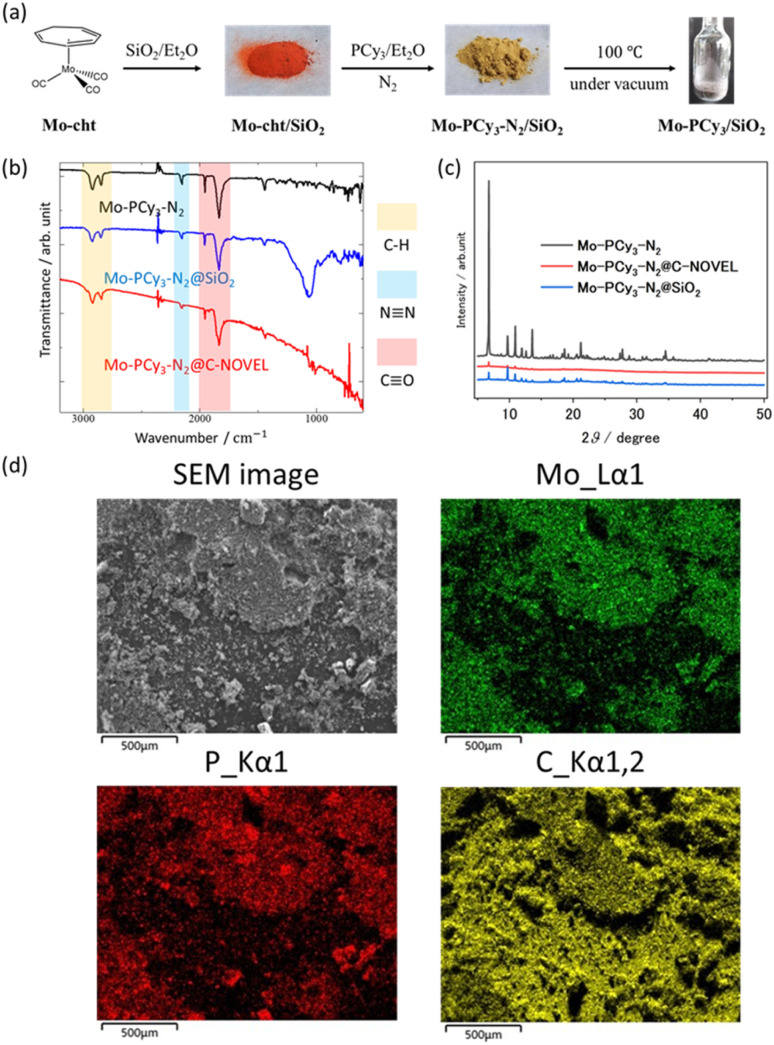
(a) Synthetic procedure of Mo-PCy_3_@SiO_2_. (b) Comparison of FT-IR spectrum. (c) Comparison of PXRD patterns. (d) SEM image and elemental mapping of Mo-PCy_3_-N_2_@C-NOVEL mounted on the carbon adhesive tape.

**Fig. 2:**
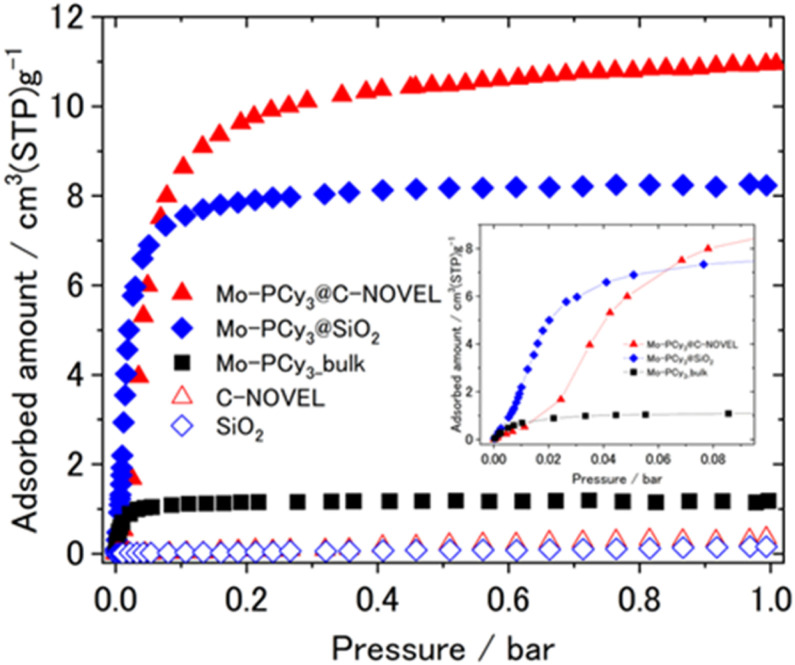
Comparison of H_2_ adsorption isotherms at 313 K.

While Fig. 2, S5 and S8† displays the hydrogen adsorption isotherms for both the bulk complex and the support materials individually, it is evident that the adsorption capacity of the supported materials exceeds the sum of the individual capacities. This suggests an efficient dispersion of the complex molecules on the support material, leading to enhanced hydrogen adsorption. PXRD and SEM/EDX measurements were conducted to investigate the dispersivity of Mo complex on porous materials. As shown in Fig. 1(c), small peaks deriving from the bulk [Mo(PCy_3_)_2_(CO)_3_(N_2_)] crystals were observed in the PXRD pattern, which indicates that the Mo complex is effectively dispersed on the support materials, although a slight presence of bulk crystals remains in the solid. The elemental mapping using SEM/EDX also show no large clumps of molybdenum and phosphorus atoms, which supports that the Mo complex molecules are uniformly dispersed on C-NOVEL. Based on these findings, we concluded that the enhancement of maximum adsorption capacities in supported complexes are attributable to the effective dispersion of complex molecules on the support materials, thereby improving accessibility to the gas molecules. The isosteric heat of adsorption analysis, depicted in Fig. S15,† reveals mostly same adsorption enthalpy between the bulk complex and the supported complexes. This result indicates that the dihydrogen complex molecules function as hydrogen adsorption sites even after supported, and the support materials had little effect on the thermodynamic properties of adsorption. However, a notable difference is observed in the shape of the adsorption isotherms. The inset in Fig. 2 shows a magnification of the adsorption isotherms up to 0.1 bar. While bulk [Mo(PCy_3_)_2_(CO)_3_] exhibits a monotonically increasing convex curve, the supported complexes display sigmoidal curves, transitioning from concave to convex. This change in the isotherm shape could be attributed to the partial filling of the pores by the complex molecules, which results in resistance to gas diffusion and behavior akin to the gate-opening phenomenon. Such a phenomenon is often observed in MOFs. Further investigation to elucidate the specifics of this phenomenon is ongoing.

To investigate the durability of the Mo complex supported on porous materials, repeated measurements of H_2_ adsorption were conducted at 80 °C. The results showed that there was no significant change in the adsorption capacity even after five cycles of adsorption and desorption, indicating that the complex exhibits high stability.

Here we discuss the effect of pore diameter of support materials. For the comparison, microporous carbon supported complex Mo-PCy_3_@MSC-30 was synthesized by using MSC-30 with the averaged pore diameter (*d*) of 18.6 Å. The detailed properties of C-NOVEL (*d* = 78.0 Å), silica gel (*d* = 58.6 Å) and MSC-30 are listed in Table S1.† The comparison of H_2_ adsorption isotherms is shown in Fig. 3(a). Although the maximum adsorption capacity of Mo-PCy_3_@MSC-30 improved to 3.6 times of bulk complex, it is much smaller than those of C-NOVEL and silica gel supported complexes. To reveal the origin of this difference, N_2_ adsorption isotherms at 77 K were measured, As shown in Fig. 3(b), C-NOVEL and silica gel supported complexes are still mesoporous with type IV isotherms albeit adsorption capacities dramatically decrease. This indicates that C-NOVEL and silica gel have large enough spaces to adsorb N_2_ molecules even after supporting Mo complexes into their pores, and therefore, the effective gas diffusion is possible. On the other hand, in the case of Mo-PCy_3_@MSC-30, no N_2_ adsorption was observed below 0.9 *P*/*P*_0_, which suggests that Mo complex molecules block the diffusion path of gas molecules, and the effect of dispersion is not as good as C-NOVEL and SiO_2_ supported complexes. This difference can be schematically depicted as Fig. 4. From these results, it can be concluded that porous materials with mesopore are more appropriate for the supporting of [Mo(PCy_3_)_2_(CO)_3_]. However, it should be noted that the ideal supporting condition could be different depending on the combination of complex and support materials, especially the sizes of the complex molecules have a significant influence.

**Fig. 3:**
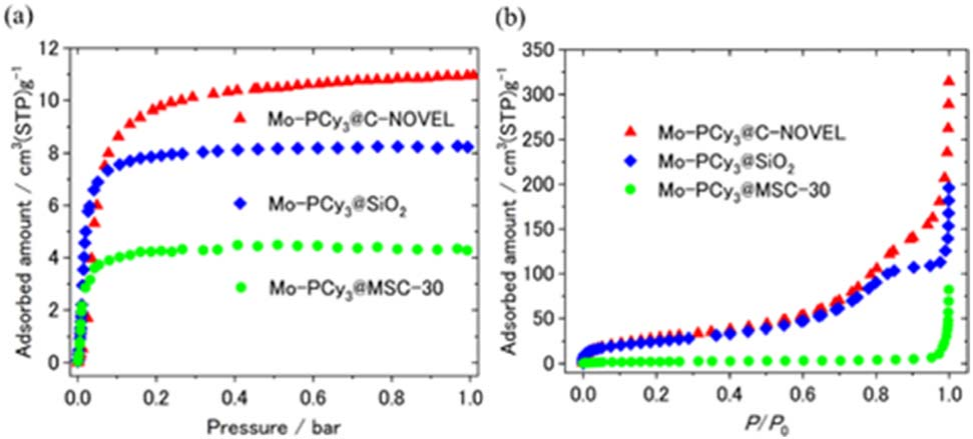
Comparison of (a) H_2_ and (b) N_2_ adsorption isotherms of supported complexes. H_2_ and N_2_ adsorption isotherms were measured at 313 K and 77 K, respectively.

**Fig. 4:**
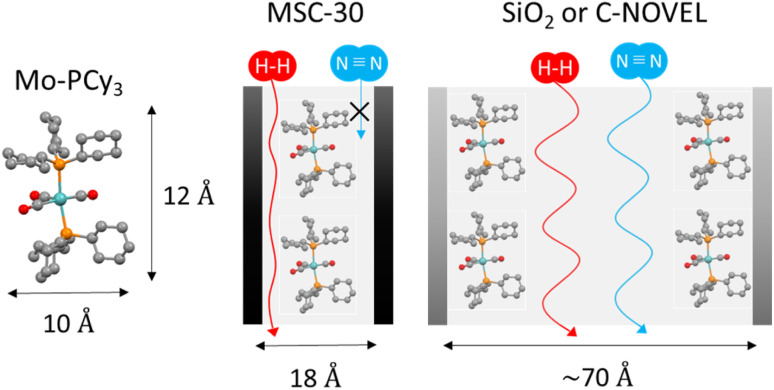
Schematical illustration of the relationship between the size of the [Mo(PCy_3_)_2_(CO)_3_] molecule and pore size of porous-material-supported complexes.

In addition, we attempted the synthesis of Mo-PCy_3_@NU-1000. NU-1000 is a Zr-oxo-cluster based MOF which has mesopore with pore diameter of >2 nm, and it could be a good support material of Mo-PCy_3_.^23^ However, contrary to our expectations, the attempt resulted in the decomposition of Mo-cht during the supporting. The decomposition of Mo-cht can be attributed to the reactive oxygen atoms or hydroxyl groups of the Zr-oxo-cluster in NU-1000. This result indicates that the chemically inert support materials should be chosen.

In this discussion, we address the loading of [Mo(PCy_3_)_2_(CO)_3_] in porous materials. From the N_2_ adsorption isotherms at 77 K for silica gel and C-NOVEL (Fig. S1†), as well as the BJH plots (Fig. S3†), the pore volumes are estimated to be 0.93 and 2.43 cm^3^ g^−1^, respectively. Meanwhile, the van der Waals volume of a [Mo(PCy_3_)_2_(CO)_3_] molecule is estimated to be 650 Å^3^ using modeling software. Based on these values, the maximum adsorption capacities are calculated to be 19.3 cm^3^ (STP) g^−1^ for silica gel and 24.9 cm^3^ (STP) g^−1^ for C-NOVEL. In our H_2_ adsorption measurements, the saturated adsorption capacities were found to be 8.2 cm^3^ (STP) g^−1^ for silica gel-supported and 11.0 cm^3^ (STP) g^−1^ for C-NOVEL-supported complexes, indicating that about 40% of the theoretical maximum adsorption capacity was achieved. Therefore, improvement in experimental conditions could further increase the saturated adsorption capacity of the complex, which remains a subject for future research. Although the H_2_ adsorption amount of the present compounds (up to 11 cm^3^ (STP) g^−1^) at 1 bar is less than that of aforementioned MOFs (36 cm^3^ (STP) g^−1^ at 203 K for Cu(i)-MFU-4l,^21^*ca.* 45 cm^3^ (STP) g^−1^ at 293 K for V(ii)-MOF,^4^ 11 cm^3^ (STP) g^−1^ at 296 K for NU-2100 (ref. 22)), it is noteworthy that this method can be applied to more than 100 existing dihydrogen complexes with various thermodynamic parameters, making it a general approach for utilizing the dihydrogen complexes as a solid material such as heterogeneous catalysts, hydrogen isotope separation, *etc.*

In conclusion, we examined the hydrogen adsorption properties of [Mo(PCy_3_)_2_(CO)_3_] when supported on silica gel and activated carbon. The results demonstrated a substantial increase in hydrogen adsorption capacity, reaching up to 9.3 times that of the bulk complex. Importantly, the thermodynamic adsorption properties remained nearly unchanged, underscoring the effectiveness of supporting dihydrogen complexes on porous materials. This straightforward approach of combining known compounds with cost-effective materials offers a promising strategy for developing superior hydrogen adsorbents for practical hydrogen storage at room temperature. The findings contribute to advancing the realization of a hydrogen-based society and promoting sustainable energy solutions. Further exploration of new material combinations and enhancements holds the potential to shape a more sustainable energy future.

## Conflicts of interest

There are no conflicts to declare.

## Supplementary Material

RA-014-D4RA01182A-s001
