# Supplementary material for: Enhanced hydrogen uptake of dihydrogen complex via porous materials support
Source: RSC Adv. 2024 Apr 9;14(16):11452–5. doi: 10.1039/d4ra01182a (PMC11003236; doi:10.1039/d4ra01182a)
Supplement: RA-014-D4RA01182A-s001 [file RA-014-D4RA01182A-s001.pdf]

Electronic Supplementary Material (ESI) for RSC Advances.
This journal is © The Royal Society of Chemistry 2024

Electric Supplementry Informations for

Enhanced Hydrogen Uptake of Dihydrogen Complex via Porous Materials Support

Kaiji Uchida,[1,2] Shuta Adachi,[3] , Shunya Tanaka,[1] Hiroaki Iguchi,[1] Ryota Sakamoto,[1] and Shinya Takaishi[1,4*]

[1]*Department of Chemistry, Graduate School of Science, Tohoku University, 6-3 Aza-aoba, Aramaki, Sendai 980-8578, Japan,*

[2]*Tokyo Metropolitan Industrial Technology Research Institute, 2-4-10 Aomi, Koto, Tokyo 135-0064, Japan*

[3]*Department of Chemistry, Facluty of Science, Tohoku University, 6-3 Aza-aoba, Aramaki, Sendai 980-8578, Japan,*

[4]*Physical and Chemical Research Infrastructure Group, RIKEN SPring-8 Center, RIKEN, Sayo, Hyogo, 679–5198 Japan*

Contents

Experimental details: 2

N_2 isotherms and BET plots of silica-gel and CNOVEL 3

Pore size distribution of silica-gel and CNOVEL 4

Variable temperature N_2 isotherms of silica-gel and CNOVEL 4

Summary of the porosity of C-NOVEL and SiO_2 4

H_2 adsorption isotherms of Mo-PCy$_3$@C-NOVEL, Mo-PCy$_3$, and C-NOVEL at 313 K 5

Variable-temperature H_2 adsorption isotherms of Mo-PCy$_3$@C-NOVEL 5

Differential isosteric heat of adsorption of Mo-PCy$_3$@C-NOVEL. 5

H_2 adsorption isotherms of Mo-PCy$_3$@SiO$_2$, Mo-PCy$_3$, and SiO_2 at 313 K 6

Variable-temperature H_2 adsorption isotherms of Mo-PCy$_3$@SiO$_2$ 6

Differential isosteric heat of adsorption of Mo-PCy$_3$@SiO$_2$ 6

H_2 adsorption isotherms of Mo-PCy$_3$@MSC-30 and Mo-PCy 313 K 7

Variable-temperature H_2 adsorption isotherms of Mo-PCy$_3$@MSC-30 7

Differential isosteric heat of adsorption of Mo-PCy$_3$@MSC-30 7

H_2 adsorption isotherms of **Mo-PCy$_3$@C-NOVEL**, **Mo-PCy$_3$**, and C-NOVEL 8

Adsorption heats of **Mo-PCy$_3$@C-NOVEL**, **Mo-PCy$_3$@SiO$_2$**.,
Mo-PCy$_3$@MSC-30 and **Mo-PCy$_3$**. 8

Repetitive H_2 adsorption measurement of **Mo-PCy$_3$@C-NOVEL** 8

EDX spectrum of **Mo-PCy$_3$@C-NOVEL** 9

Experimental Details

Synthesis of [Mo(η^6-1,3,5-cycloheptatriene)(CO)$_3$] (Mo-cht)

Nitrogen-purged 2-necked flask was charged with molybdenum hexacarbonyl $Mo(CO)_6$ (4.0 g, 15.15 mmol), 1,3,5-Cycloheptatriene (9.0 ml, 86.74 mmol) and 40 ml of N_2-saturated heptane. The white suspension was refluxed at 130 °C for 24 h and cooled down to room temperature, which gave red solution and red precipitate. The reaction mixture was filtered and washed with heptane. Residual red solid was extracted with hexane and combined with red heptane solution. Solvent was removed by rotary evaporator and resultant red solid was subject to vacuum sublimation at 40 °C to remove the unreacted $Mo(CO)_6$, which gave analytically pure red crystalline solid of target compound.

Yield: 86 %

Elemental analysis: Calculated for MoC10H8O3 C: 44.141 H: 2.963 N: 0.000

Found C: 43.761 H: 2.963 N: 0.000

Synthesis of Mo-PCy$_3$@C-NOVEL

Round-bottomed flask was charged with **Mo-cht** (272 mg 1.0 mmol), and diethyl ether (10 ml) was added to obtain the clear reddish orange solution. To this solution, C-NOVEL-MH (100 mg) was added and shook slightly to make a homogeneous black suspension, and solvent was removed by rotary evaporator. Diethyl ether was added to the residual black powder, and solvent was removed again to get the uniformly dispersed **Mo-cht**@C-NOVEL. Tricyclohexylphosphine (574 mg, 2.05 mmol) was added to this black powder, and the vial was transferred to N_2-glovebox. N_2-saturated diethyl ether (4.0 ml) was added and stirred at room temperature. After 4 h, diethyl ether (10 ml) was added and stand overnight. The bulk crystals of $Mo(PCy_3)_2(CO)_3N_2$ were removed by filtration with coarse filter paper and then black precipitate was filtered and washed with diethyl ether for several times, and dried under vacuum to get the title compound.

Synthesis of Mo-PCy$_3$@SiO$_2$

Same synthetic procedure was used as **Mo-PCy$_3$@C-NOVEL,** and the title compound was obtained as yellow powder.

Measurements

H_2 and N_2 sorption isotherm was performed using microtracbel Belsorp Max. IR spectra were recorded as KBr pellets on a JASCO FT/IR-4200 spectrometer at room temperature. For acquiring IR spectra under inert atmosphere, the KBr pellets were fabricated in glove box (MBRAUN UNILAB1200/780) filled with Ar gas, and then the pellets were set into a specially designed sealed optical cell individually. Elemental analyses were performed using J-Science Lab JM-10 equipped in the research and analytical center for giant molecules at Tohoku University. SEM/EDX measurements were performed using JEOLJSM-7800F combined with Oxford instruments X-MAX 50.

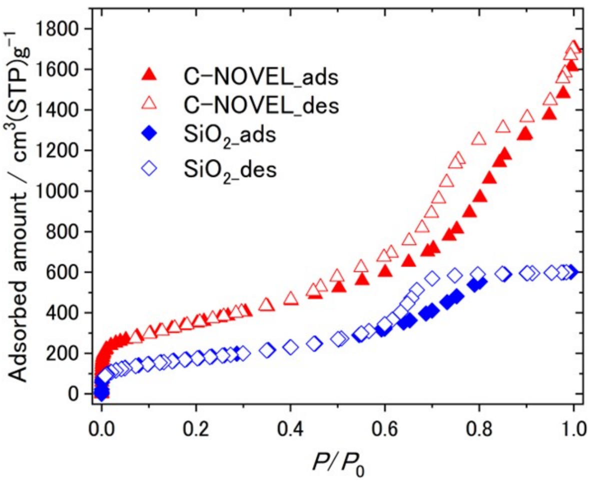

Figure S1 N_2 sorption isotherms of C-NOVEL and SiO_2 at 77 K. Filled symbol represents adsorption data and hollow symbol represents desorption data.

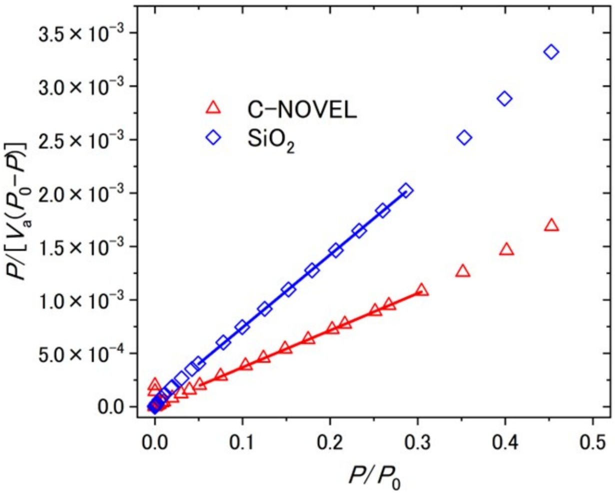

Figure S2 BET plots made from N_2 sorption isotherms of C-NOVEL and SiO_2.

□□

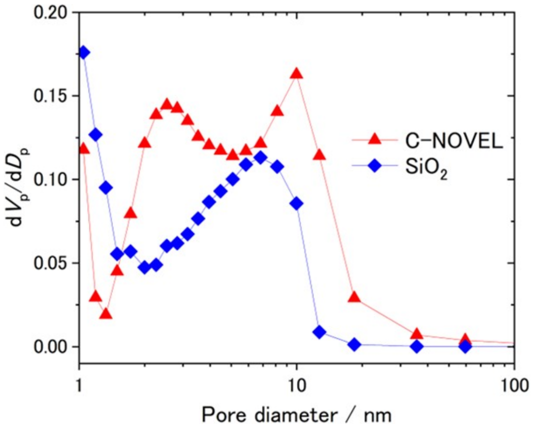

□□□□

Figure S3 Pore size distribution of C-NOVEL and SiO_2. The plots were made by from BJH method.

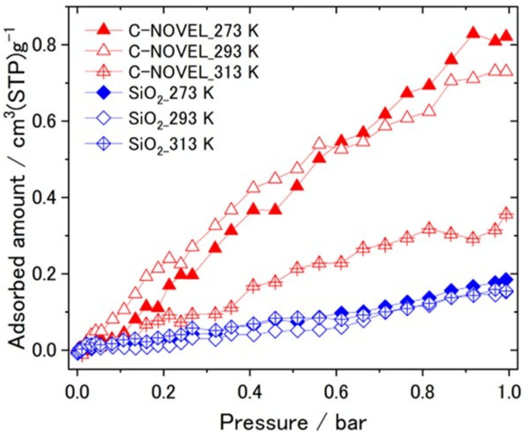

□□□□□□□□

Figure S4 Variable-temperature H_2 adsorption isotherms C-NOVEL and SiO_2.

Table S1 Properties of the porous materials used in this study

	C-NOVEL	SiO_2	MSC-30*
BET surface / m^2 g^{-1}	1248.9	633.9	3200
Pore volume / cm^3 g^{-1}	2.43	0.929	1.65
Averaged pore diameter / nm	7.80	5.86	1.86
H_2 adsorption amount / cm^3 g^{-1} at 273 K	0.82	0.18	-

*The data of MSC-30 were quoted from the catalogue.

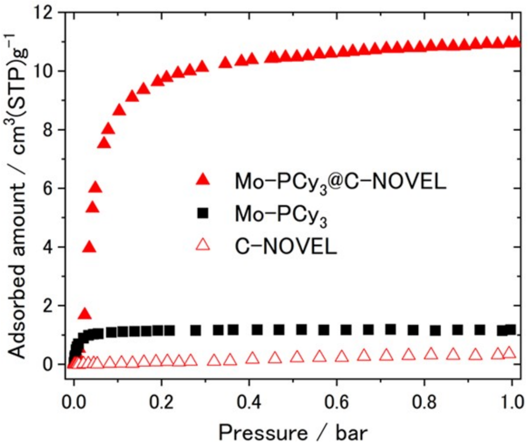

[illegible] [illegible]

Figure S5 Comparison of H_2 adsorption isotherms of **Mo-PCy$_3$@C-NOVEL**, **Mo-PCy$_3$**, and C-NOVEL.

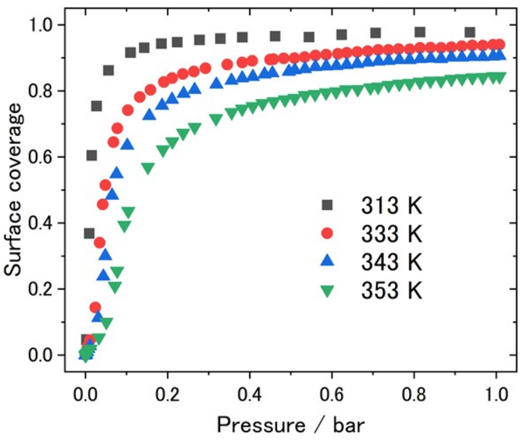

Figure S6 Variable-temperature H_2 adsorption isotherms of **Mo-PCy$_3$@C-NOVEL**.

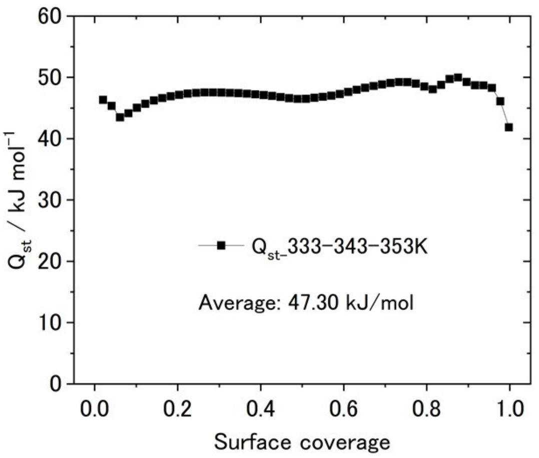

[illegible] [illegible]

Figure S7 Differential isosteric heat of adsorption of **Mo-PCy$_3$@C-NOVEL**.

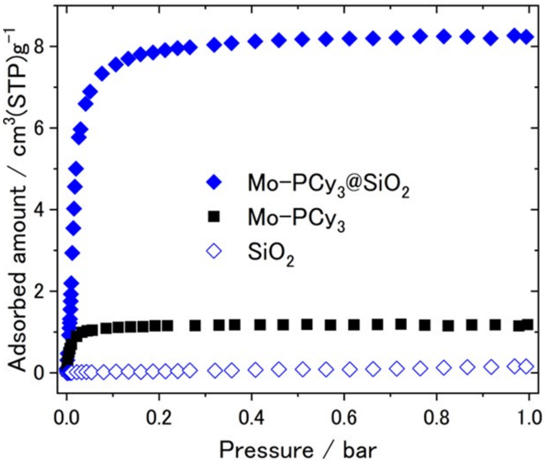

□□□□□□□□□□

Figure S8 Comparison of H_2 adsorption isotherms of **Mo-PCy$_3$@SiO$_2$**, **Mo-PCy$_3$**, and SiO_2.

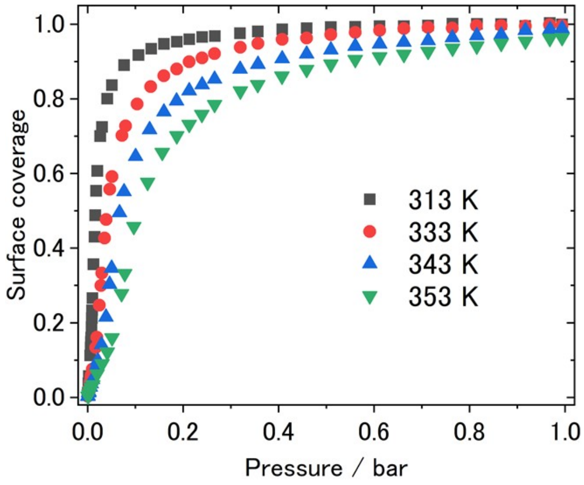

Figure S9 Variable-temperature H_2 adsorption isotherms of **Mo-PCy$_3$@SiO$_2$**.

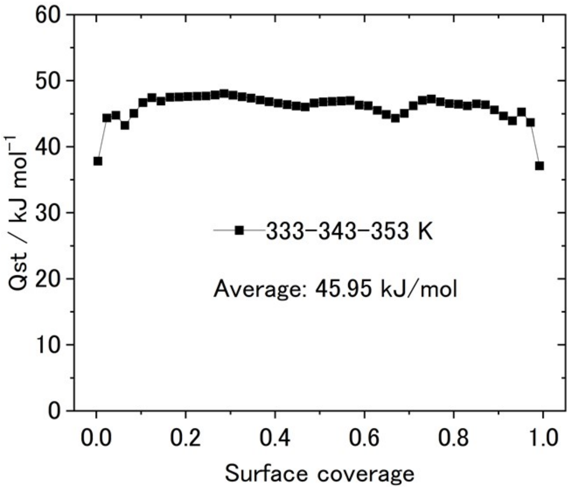

Figure S10 Differential isosteric heat of adsorption of **Mo-PCy$_3$@SiO$_2$**.

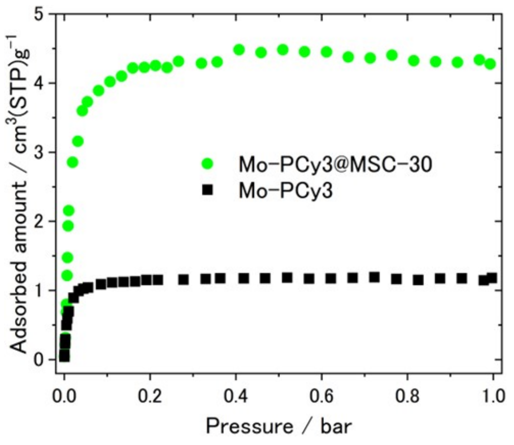

Figure S11 Comparison of H_2 adsorption isotherms of **Mo-PCy$_3$@MSC-30**, **Mo-PCy$_3$**, and MSC-30.

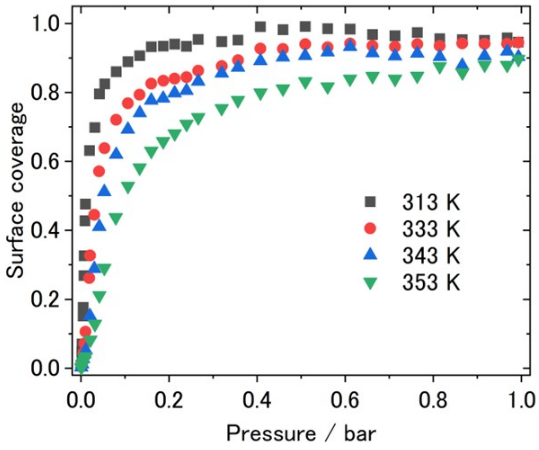

Figure S12 Variable-temperature H_2 adsorption isotherms of **Mo-PCy$_3$@MSC-30.**

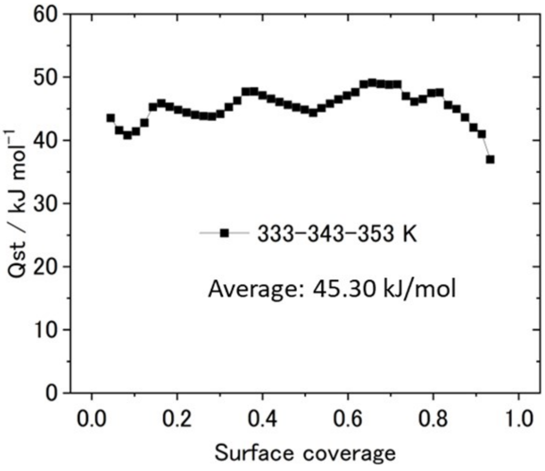

Figure S13 Differential isosteric heat of adsorption of **Mo-PCy$_3$@MSC-30.**

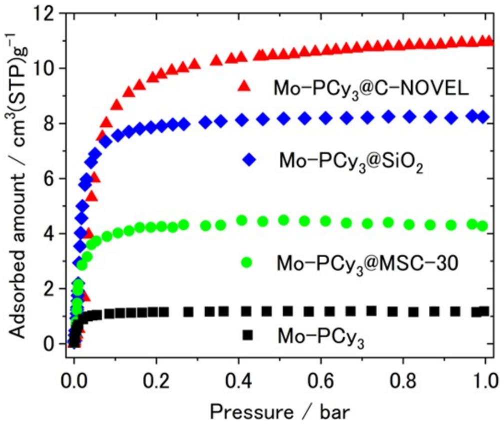

Figure S14 Comparison H_2 adsorption isotherms of **Mo-PCy$_3$@C-NOVEL**, **Mo-PCy$_3$@SiO$_2$**., **Mo-PCy$_3$@MSC-30** and **Mo-PCy$_3$**.

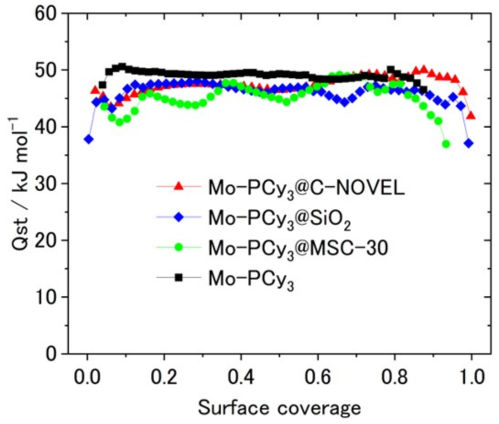

Figure S15 Comparison of adsorption heats of **Mo-PCy$_3$@C-NOVEL**, **Mo-PCy$_3$@SiO$_2$**., **Mo-PCy$_3$@MSC-30** and **Mo-PCy$_3$**.

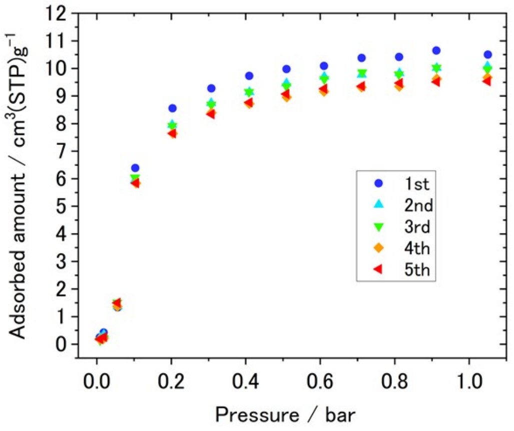

Figure S16 Repetitive H_2 adsorption measurement of **Mo-PCy$_3$@C-NOVEL** at 80°C.

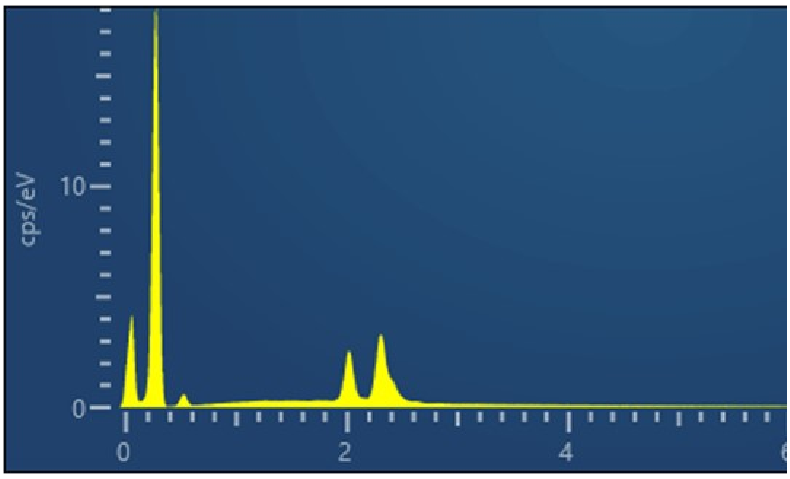

Figure S17 SEDX spectrum of **Mo-PCy_3@C-NOVEL**.
